# Phantom Validation of a Conservation of Activity-Based Partial Volume Correction Method for Arterial Input Function in Dynamic PET Imaging

**DOI:** 10.3390/tomography8020069

**Published:** 2022-03-21

**Authors:** Brandon Driscoll, Tina Shek, Douglass Vines, Alex Sun, David Jaffray, Ivan Yeung

**Affiliations:** 1 Quantitative Imaging for Personalized Cancer Medicine (QIPCM)—Techna Institute, University Health Network, Toronto, ON M5G 2C4, Canada; tina.shek@uhn.ca (T.S.); dasjaffray@mdanderson.org (D.J.); ivan.yeung@uhn.ca (I.Y.); 2 Radiation Medicine Program, Princess Margaret Cancer Centre, Toronto, ON M5G 2M9, Canada; doug.vines@uhn.ca (D.V.); alex.sun@uhn.ca (A.S.); 3 Department of Radiation Oncology, University of Toronto, Toronto, ON M5T 1P5, Canada

**Keywords:** dynamic PET, kinetic modelling, partial volume correction, cancer imaging, quantitative imaging

## Abstract

Dynamic PET (dPET) imaging can be utilized to perform kinetic modelling of various physiologic processes, which are exploited by the constantly expanding range of targeted radiopharmaceuticals. To date, dPET remains primarily in the research realm due to a number of technical challenges, not least of which is addressing partial volume effects (PVE) in the input function. We propose a series of equations for the correction of PVE in the input function and present the results of a validation study, based on a purpose built phantom. ^18^F-dPET experiments were performed using the phantom on a set of flow tubes representing large arteries, such as the aorta (1” 2.54 cm ID), down to smaller vessels, such as the iliac arteries and veins (1/4” 0.635 cm ID). When applied to the dPET experimental images, the PVE correction equations were able to successfully correct the image-derived input functions by as much as 59 ± 35% in the presence of background, which resulted in image-derived area under the curve (AUC) values within 8 ± 9% of ground truth AUC. The peak heights were similarly well corrected to within 9 ± 10% of the scaled DCE-CT curves. The same equations were then successfully applied to correct patient input functions in the aorta and internal iliac artery/vein. These straightforward algorithms can be applied to dPET images from any PET-CT scanner to restore the input function back to a more clinically representative value, without the need for high-end Time of Flight systems or Point Spread Function correction algorithms.

## 1. Introduction

The vast majority of Positron Emission Tomography (PET) studies involve the imaging of static, late whole-body images, which are typically done between 60 [1,2,3,4,5,6] and 90 [2,3,4,5,7] minutes post injection. These scans produce images which can be converted to units of SUV (Standardized Uptake Value), based on the injected radiotracer dose, half-life, time from injection and patient weight. The SUV values of a region of interest (ROI), especially noise-dependent values, such as the max SUV (SUV_max_), are dependent on a wide range of factors (scanner calibration, reconstruction algorithm, frame duration, scatter correction, presence of Time of Flight capability, etc.) and can be difficult to compare between systems and institutions [1,5]. More importantly, the SUV of a static scan is a snapshot of the underlying dynamic processes, which, as a whole, may provide a wealth of information about a patient’s underlying physiology.

One intrinsic benefit of PET is that the dynamic imaging (i.e., imaging the patient continuously) can be performed over the course of minutes and up to an hour, without additional radiation dose to the patient. Kinetic modeling with dynamic imaging data can help describe important physiologic processes, such as regional blood flow, glucose metabolism or receptor density [1,2,3,4,5,8,9]. Although dynamic PET (dPET) imaging is increasingly applied with different radiotracers to measure various organs or disease physiology, it has not reached widespread clinical usage [1,6].

The review article by Dimitrakopoulou-Strauss et al. (2021) [1] performed a detailed literature search of over 130 papers on compartment modelling in dPET, with the most commonly used tracer being a glucose analog,^18^F-FDG (n = 42). Over 30 different radiotracers have been investigated within this volume of work, including agents aimed at binding to receptors over-expressed on certain cancer cells, such as ^18^F-PSMA or ^68^Ga-PSMA and ^68^Ga-DOTA [10,11,12,13,14,15], while other radiotracers assess other metabolic processes, such as hypoxia, using ^18^F-FMISO, ^18^F-FAZA or ^18^F-HX-4 [16,17,18,19,20,21,22].

To perform quantitative studies using dPET, some form of mathematical model describing the radioligand kinetics is required [1,5,8,9]. Typically, these kinetic models include an “arterial input function” (AIF), representing the image- or population-derived amount of tracer in the arterial blood, leading to an ROI (i.e., a function of time) and the ‘‘time activity curve’’ (TAC), which is the image-derived radioactivity in the tissue ROI over time. These two sets of data are acquired during the dynamic acquisition, and they are fitted into the kinetic model to estimate the associated model parameters.

Unfortunately, the limited axial field of view (FOV) of standard PET-CT scanners is typically in the range of 15–20 cm, which precludes measuring multiple target lesions at once. This small FOV also prevents the sampling of an arterial input function from a large artery, such as the aorta (ID > 2 cm), when the lesion or organ of interest is located outside the thorax. As a result, the image-based input function for kinetic models often must be sampled using much smaller (ID < 1 cm) arteries (internal carotid for brain, internal iliac for prostate, rectum or cervix).

**Figure 1:**
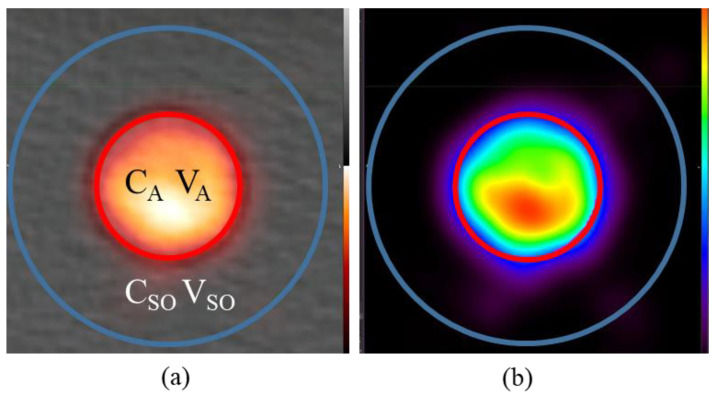
Visualization of PET PVE in the presented phantom, specifically the “spill-out” effect from the hot to cold region. (**a**) The true artery ROI (outlined in red) as contoured in CT is listed as having image-derived concentration C_A_ and volume V_A_ while the large region (labelled C_SO_/V_SO_, outlined in blue) is comprised of the true hot region plus the “spill-out” region. (**b**) Visualization of the “spill-out” from the V_A_ region into V_SO_ shown on the PET only.

This limitation results in challenges in performing kinetic modelling, because in order to obtain accurate kinetic model parameters, the input function must not be affected by partial volume effects [1,5]. Partial volume effects (PVE) in PET occur as a result of the low spatial resolution of PET imaging (4–6 mm) [5]. As a result of the limited spatial resolution, small objects can have much of their activity “spilled-out” of the actual region (Figure 1), such that the mean activity of the contoured region is underestimated. When the objects are sufficiently small (<2–3 times the spatial resolution of the system, which is typically 4–7 mm), even the SUV_max_ may be significantly reduced [6]. Unfortunately, small vessels, such as the internal iliac or femoral artery, typically have diameters between 5 and 8 mm, which leads to their image-derived AIF being substantially reduced, causing inaccuracies in kinetic modelling results.

A number of correction strategies have attempted to address PVE of AIF in dPET. The most common approach applies the use of recovery coefficients, which is a typical approach applied in static PET scans. This approach involves performing a relative correction, using a constant for a region based on its true size [1,5,23,24,25,26,27]. These approaches are primarily designed for spherical lesions and intended to correct only the SUV_max_ (a measurement of 1 voxel) of a region of interest, leaving the SUV_mean_ unaccounted for. Unfortunately, the SUV_max_ is more sensitive to statistical fluctuations, especially in situations with very high PET noise [5,28]. High noise is the norm in dPET, where frame duration can be as low as 10 s, and this often leads to an overestimation of the true value [28]. Another challenge to these approaches corrects only for the “spill-out” from the ROI, while neglecting the “spill-in” from other regions into the ROI, from either the background or another high-activity region (veins, bladder, etc.).

Many modern PET-CT systems also include point-spread function (PSF) correction algorithms, which have been applied to correct AIF [29] in dynamic studies. These PSF algorithms attempt to computationally correct the SUV_max_ of lesions via iterative deconvolution approaches. These approaches occur right on the system, so they are easy to perform; however, they often result in overcorrection [30] and, as in the case of recovery coefficients, really only work at correcting the noise-dependent SUV_max_ term [28]. 

In a properly calibrated PET-CT scanner, the total activity should always be accounted for somewhere in the image field of view (FOV), such that even when there are partial volume effects, the cumulative activity in the image is still correct [5,31]. In simplified terms, the observed signal in an ROI is made up of the actual activity minus the “spill-out” to other regions, plus the “spill-in” from other neighbouring regions to the ROI. The use of recovery coefficients is based on this theory and utilizes a set of known coefficients, derived from spherical phantom measurements, to determine the true activity for a sphere, based on the measured activity and the volume of the sphere. In this manuscript, we propose a straightforward approach, based on the conservation of activity, to account for the activity “spilled-in” to an ROI from the background, as well as that “spilled-out” of the ROI.

Though simple, this approach involves different equations for different scenarios that need thorough validation before they can be utilized for AIF correction in patient studies [32]. To validate these correction equations, a phantom was designed and constructed with the capability of generating a set of input functions on different diameter tubes but maintaining the input function shape (both peak intensity and area under curve, AUC). 

## 2. Methods

### 2.1. Partial Volume Effect Correction via Conservation of Activity

We propose a numerical approach, based on the conservation of activity (CoA), to correct the mean activity in a region by adding all the activity in a larger region which includes the “spill-out” and dividing by the true volume as shown in Equation (1).

(1) $$C_{A\;Corr}=\frac{C_{SO}V_{SO}}{V_{A}}$$

In this equation C_A Corr_ represents the *corrected* activity (C_A_) in Bq/cc in the defined tube/artery region V_A_ (in cubic centimeters (cc)) while C_A_ is the image-derived activity if simply reporting the mean activity within V_A_ without correction. V_A_ can be accurately measured on the CT image for a PET-CT scanner. This C_A Corr_ value is obtained by multiplying the larger mean activity of the tube region which includes the “spill-out” (C_SO_) by the larger volume (V_SO_) then dividing by the true tube/artery volume (V_A_). Note that C_SO_ and V_SO_ encompass the C_A_ and V_A_ respectively. V_SO_ is also assumed to be large enough to ‘contain’ all the ‘spill-out’ signal from tube.

**Figure 2:**
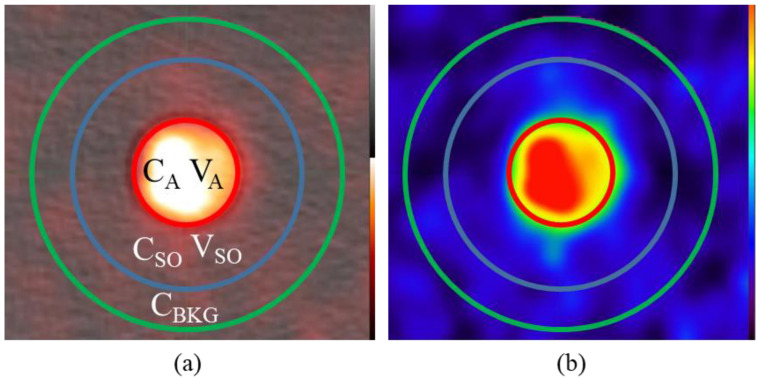
Partial volume correction in the presence of background. (**a**) In addition to C_A_/V_A_ and C_SO_/V_SO_ an additional region outside of the “spill-out” region is selected as the background concentration C_BKG_ is outlined in green. (**b**) Visualization of the “spill-out” from the V_A_ region into V_SO_ is still evident even in the presence of background shown on the PET only.

This simple approach in Equation (1) only works in regions where the background concentration is zero. As observed in clinical scans, there is always a low level of residual activity throughout the patient even after a few hours post radiotracer injection. In order to incorporate background into Equation (1), we assume the region outside V_A_ but within V_SO_ is filled with background activity in addition to ‘spill-out’ signal from the V_A_ as displayed in Figure 2. Therefore, the measured activity in V_A_ will require subtraction of background activity in the annulus region of V_SO_ to correctly capture the ‘spill-out’ signal.

(2) $$C_{A\;Corr}=\frac{C_{SO}V_{SO}-C_{BKG}\left(V_{SO}-V_{A}\right)}{V_{A}}$$

where C_BKG_ is the concentration in annulus region between the blue and green circle in Figure 2. Its location is assumed to be far enough from any ‘spill-out’ signal from the tube and therefore C_BKG_ represents the ‘true’ background concentration. It is also assumed that the amount of “spill-in” from the background to V_SO_ is equal to the “spill-out” from V_SO_ to the background which should be true provided V_SO_ is sufficiently large.

**Figure 3:**
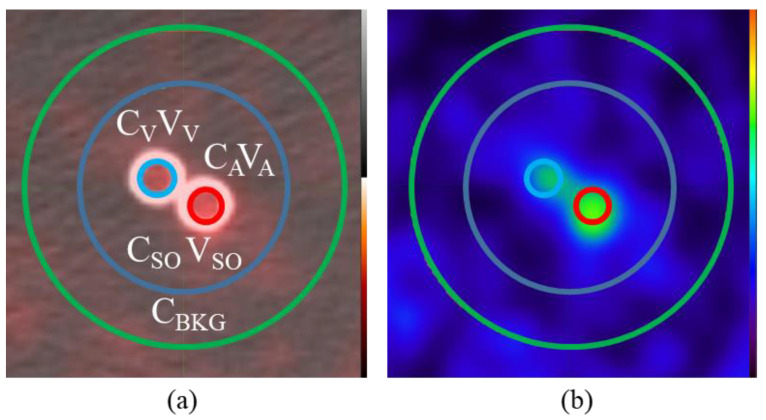
Partial volume correction in the presence of two signal functions. (**a**) In addition to the “spill-out” (C_SO_/V_SO)_ and background activity (C_BKG_), the C_A_/V_A_ terms for the arterial input as well as new terms C_V_/V_V_ representing the venous function are separately outlined. (**b**) “Spill-out” from these small ¼” tubes is easily observable on the PET image.

In situations where there are other vessels in the region of the AIF, such as the case of the internal iliac artery and veins, additional corrections and assumptions are required. In this scenario, depicted in Figure 3, both the artery and vein contribute to the activity in V_SO_.

In order to address this combined influence on the cumulative activity in the “spill-out” we can include both the C_A CORR_ and C_V CORR_ terms in the activity balance and make sure we also subtract the area of both vein V_V_ and artery V_A_ from the background contribution. We need to make another assumption that the contribution of the total activity in the artery and vein are proportional to the image-derived signal C_A_V_A_ and C_V_V_V_. Making those assumptions leads to the derivation of Equation (6) below.

(3) $$C_{A\;Corr}V_{A}+C_{V\;Corr}V_{V}=C_{SO}V_{SO}-C_{BKG}\left(V_{SO}-V_{A}-V_{V}\right)$$

Assume:

(4) $$\frac{C_{A\;Corr}V_{A}}{C_{V\;Corr}V_{V}}=\frac{C_{A}V_{A}}{C_{V}V_{V}}$$

Sub B into A:

(5) $$C_{A\;Corr}V_{A}+\frac{C_{A\;Corr}V_{A}C_{V}V_{V}}{C_{A}V_{A}}=C_{SO}V_{SO}-C_{BKG}\left(V_{SO}-V_{A}-V_{V}\right)$$

(6) $$C_{A\;Corr}=\frac{C_{SO}V_{SO}-C_{BKG}\left(V_{SO}-V_{A}-V_{V}\right)}{V_{A}}\times \frac{C_{A}V_{A}}{C_{A}V_{A}+C_{V}V_{V}}$$

### 2.2. Phantom Design

**Figure 4:**
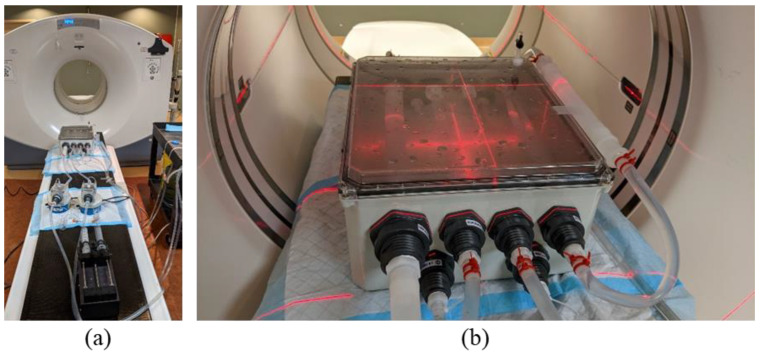
The AIF Correction factor validation phantom. (**a**) The four tubes inside the phantom shell decreasing in size from 1” to 1/2”, 3/8” and finally 1/4” followed by the buffer tube at the right side. (**b**) The injection setup on the PET-CT couch. Scanning is performed in the middle of the tubes over a single (14.7 cm) PET field of view.

To test and validate PVE correction models for dynamic PET-CT, we required a phantom capable of creating an identical input function across multiple tube diameters (Figure 4). The phantom was created with a flow system similar in design to that of Driscoll et al. [33] but the perfusion chamber was replaced with the novel phantom shell and tubes to serve as background and hot regions, respectively.

This phantom contained a water-tight shell as well as a set of 4 tubes of decreasing diameter from 1” (25.4 mm) to 1/4” (6.35 mm). The choice of these tube sizes was to resemble the size of descending aorta (1”) to that of the internal iliac arteries [5] which are typically used as the AIF for numerous perfusion sites including cervix, prostate and rectum. The 1/4” tube then left the phantom and was diluted with a buffer compartment before re-entering the phantom to simulate venous flow from the iliac vein.

In addition to the input function tubes, a flow system was attached to fill and inject contrast/radioisotope into the background compartment. Flow @ 10 mL/s was perfused through the tubes using a peristaltic pump (Fisher Scientific, Hampton, NH, USA) with control valves allowing for flow through the tubes, background or both simultaneously.

### 2.3. Experimental Setup and Validation in CT

To establish reference ‘input function’ curves with consistent peak activity and AUC, dynamic contrast enhanced (DCE)-CT Scanning was performed with a 1 s Gantry rotation 120 kV, 350 mA every 6 s for 4 min on a GE Discovery 610 PET-CT system. 

The phantom was set up on the CT couch as shown in Figure 4. A Harvard Syringe Injection Pump was attached to the system and connected to each of the two mixing chambers, one for the input function and one for background. A 60 cc syringe was loaded with a 1:1 mixture of 370 mgI/mL Iodine based contrast agent and water. The flow rate through the system was set to 600 mL/min. Injection of the contrast agent occurred at 0.5 mL/s for 50 s. A replicate experiment was performed to produce error bars (range of measurements n = 2). 

### 2.4. PET-CT Experiments

Every attempt was made to ensure that the PET-CT protocol generated identical time activity curves to the DCE-CT scan. The same setup was utilized for the PET-CT experiments with the only difference being that instead of a 1:1 mix of 370 mgI/mL contrast, we used 88 MBq ^18^F-FDG diluted in 100 cc water. From the ^18^F-FDG dilution, 60 cc (53 MBq) was loaded into a syringe to be injected @ 0.5 mL/s for 50 s as in DCE-CT. For the background compartment, a separate syringe was loaded with 30 MBq ^18^F-FDG diluted in water.

Two PET-CT experiments were performed; one with background and one without. Background ^18^F-FDG was injected through the second mixing chamber between the first and second experiment providing physiologically relevant background level of 1.35 kBq/cc. 

Dynamic PET-CT was acquired in 15 × 20 s bins on the same GE Discovery 610 system (256 × 256 matrix size 3.27 mm slice thickness). This system does not have Time of Flight capability and no point spread function-based corrections were applied to the images. The area under the curve (AUC) of the PET-CT experiments was calculated based on the sum of the mean ROI activity at each time point, multiplied by the bin width (20 s), for units of kBq x s/cc. The ground truth AUC for comparison is based on the known injected activity of the PET-CT injected over the 50 s period and decay corrected to the time of experiment. Attenuation correction for the dynamic PET was based on a single low-dose static CT taken prior to radioisotope injection.

### 2.5. Image Analysis and Partial Volume Correction

DICOM images were analyzed in MIM 6.8.6 (MIM Software Inc., Cleveland, OH, USA) where 3D cylindrical ROIs were placed along the length of the tubes at the actual tube size (with activity C_A_). A second region (with activity C_SO_) was created with a radius 1.5 cm larger than V_A_. For scans with background an additional region (with activity C_Bkg_) was added as a ring with inner radius the same as C_SO_ and outer radius 1 cm larger (Figure 3). For the 1/4” vein and artery tubes, a combined C_SO_ region was contoured using a 2 cm diameter cylinder encompassing both tubes and their “spill-out”. Due to the relative complexity of the scan, only the two experiments were performed, however, multiple ROIs (n = 5) were created along the tube with similar axial dimensions (2 cm each) to the amount of straight internal iliac artery available. Error bars were derived from these measurements (±Standard Error n = 5).

In order to directly compare PET-CT and DCE-CT curves on the same graph in terms of shape and magnitude, it is necessary to establish conversion units for PET-CT and DCE-CT. Unit of HU can be converted to mgI/mL by dividing the contrast to enhancement ratio of 23 HU/mgI/mL @ 120 kVp based on a contrast calibration phantom scan. Based on this conversion factor and the total injected amount of ^18^F (22 MBq) and iodine (4625 mg), a ratio of 4.8 HU/kBq/cc was obtained and applied to scale the PET-CT and DEC-CT curves for plotting on the same graph. 

### 2.6. Application of the Input Function Correction Model to Sample Patient Image Sets

dPET DICOM images from two patients were obtained as part of two separate REB-approved research studies (NCT02701699 and NCT01567800) using 18F-FAZA. Informed consent was obtained as part of study recruitment for both patients. One scan involved a 20 min dynamic scan of the thorax of a patient of which the aorta was measured while an additional patient had a 20 min dynamic scan of their prostate in which the internal iliac artery and vein could be measured. In the first case Equation (2) was applied to the aorta and in the second case Equation (6) was applied to the artery and vein.

## 3. Results

### 3.1. DCE-CT Results and Validation

**Figure 5:**
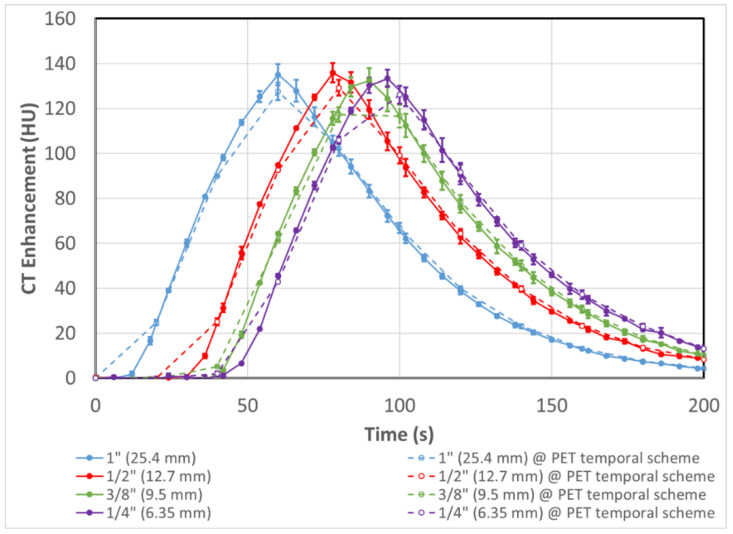
Solid lines are input function shape and intensity of the 4 tubes of decreasing internal diameter as assessed with DCE-CT. As the contrast passes through tubes of decreasing size the velocity of the fluid increases but the flow rate remains constant which produces a set of 4 reference curves with equal intensity, shape and AUC. Dashed lines are curves with averaging done over 20 s on the corresponding solid lines at the same sampling time points as the dPET scan (described in Section 3.2). Error bars represent the experimental range of replicate results (n = 2).

Due to the design of the phantom, with the size of the tubes shrinking from 1” down to 1/4” ID, neither the peak intensity nor shape of the input functions were expected to change from tube to tube. As the fluid moves through the system, the velocity increases but the volumetric flow rate remains constant. As a result, the reference curves generated maintained the same peak height, shape and AUC (Figure 5). The peak intensities of all 4 curves were within 0.6% of the group mean, while the curve AUCs were within 1.6% of the group mean.

For all intents and purposes, the DCE-CT curves sampled the input curves instantaneously, while the dPET scans were done with a 20 s bin time. Therefore, the ‘instantaneous’ CT curves were averaged with the same temporal scheme as the dPET scan to produce a set of averaged DEC-CT curves (dashed lines in Figure 5), as a reference for comparison in subsequent figures. 

### 3.2. PET-CT Results and Corrections—Without Background

**Figure 6:**
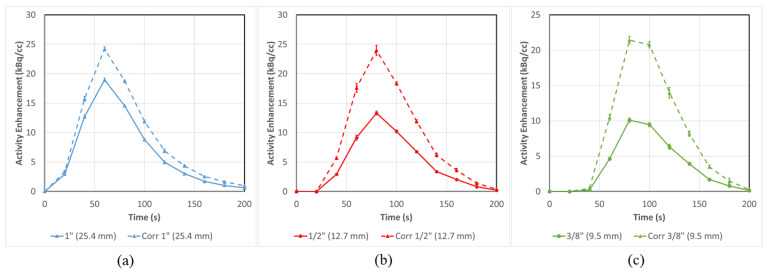
Mean input function activity can be successfully corrected for flow tubes (**a**) 1” (25.4 mm), (**b**) ½” (12.7 mm) and (**c**) 3/8” (9.5 mm) in a situation with no background. All three tubes are corrected to varying degrees with smaller tubes having larger corrections (42 ± 4% for 3/8”) than the large ones (21 ± 3% for 1”) in terms of AUC Error bars represent the standard error from multiple ROI regions (n = 5).

A dynamic PET-CT scan was performed using the same injection and flow parameters as for DCE-CT, except substituting ^18^F-FDG for contrast. No radioactivity was injected into the background compartment in this experiment. As observed in Figure 6 (solid lines), significant PVE is evident, and the measured peak activity becomes progressively depressed as the tube size becomes smaller. Only the largest three tubes are displayed in Figure 6, as the presence of the neighbouring 1/4” venous flow tube requires correction with Equation (6) (see Section 3.4). By applying Equation (1), we can correct the PVE by assigning all the “spill-out” activity in the regions surrounding the tube to the tube itself.

The correction equation results in an absolute increase in the AUC of 21 ± 3%, 39 ± 4% and 42 ± 4% for the 1”, 1/2” and 3/8” tubes, respectively. All quantitative results for all the validation studies are summarized in Table 1.

### 3.3. PET-CT Results and Corrections (with Background)

**Figure 7:**
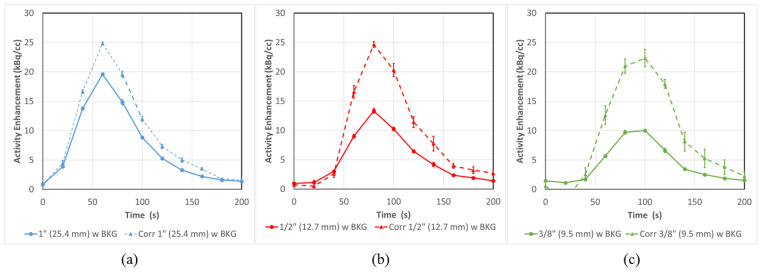
PVE in dPET Input functions at all tube sizes (**a**) 1” (25.4 mm), (**b**) ½” (12.7 mm) and (**c**) 3/8” (9.5 mm) can be substantially corrected even in conditions of background activity. The substantial PVE observed in the uncorrected (solid lines) input function can be corrected (dashed lines) by as much as 49 ± 15% for the 3/8” tube based on AUC. Error bars represent the standard error from multiple ROI regions (n = 5).

The same experiment as in Section 3.2 was repeated after 30 MBq of activity was injected into the background compartment. This provided a background activity of 1.4 kBq/cc, which is comparable to typically observed background concentrations in our dPET research scans in patients. Similar to Figure 6, Figure 7 (solid lines) shows that PVE becomes more evident as the tube size decreases. 

In this case, Equation (2) must be applied to correct the input functions to more representative values, due to the presence of background. As in Figure 6, these corrected curves (Figure 7 dashed lines) are brought roughly in line with each other as the AUCs are recovered by 21 ± 4%, 38 ± 9% and 49 ± 15% for the 1”, 1/2” and 3/8” tubes, respectively.

**Figure 8:**
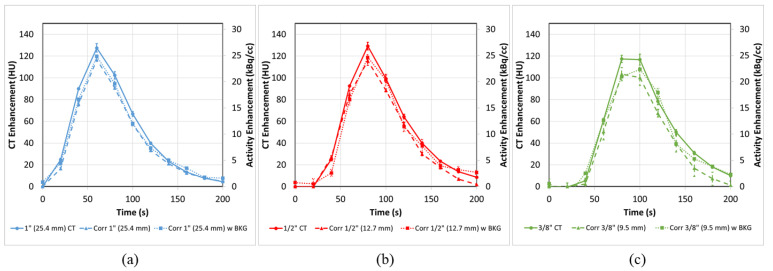
Corrected input functions at all tube sizes (**a**) 1” (25.4 mm), (**b**) ½” (12.7 mm) and (**c**) 3/8” (9.5 mm) with (dotted) and without (dashed) background are restored to various degrees in terms of both peak height and AUC. In the presence of background all three tubes have calculated AUC within 8 ± 9% of ground truth and peak heights within 9 ± 7% of averaged DCE-CT. In the absence of background these values are all somewhat reduced. PET-CT error bars represent the standard error from multiple ROI regions (n = 5). CT error bars represent the experimental range of replicate experiments (n = 2).

When comparing the PET-CT curves (Figure 6 and Figure 7) to the predicted DCE-CT curves from Figure 5, we observe good alignment in both shape and intensity for the three curves (Figure 8). The CT and PET axis are adjusted based on a calculated scaling factor of 4.8 HU/kBq/cc.

**Figure 9:**
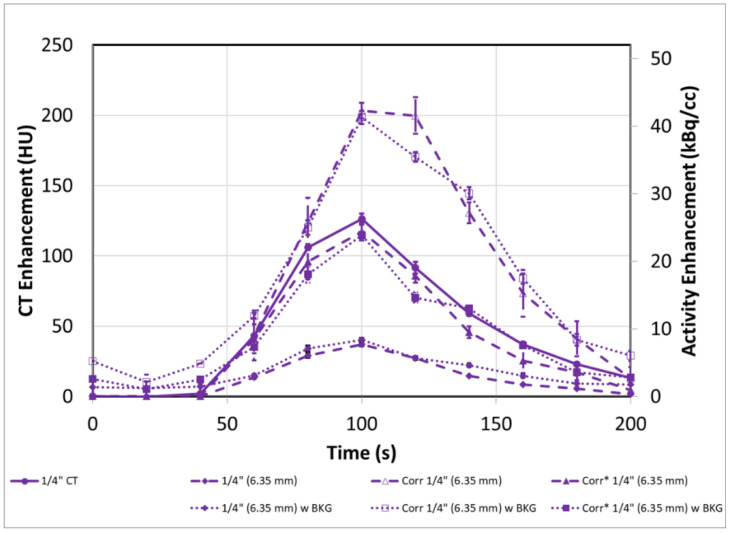
The overcorrection (open symbols) of the original input functions (filled circles) due to proximity of the venous tube when using Equation (2) (diamond symbols) can be corrected (Cor*) using Equation (6) (closed symbols) in both cases with and without background. PET-CT error bars represent the standard error from multiple ROI regions (n = 5). CT error bars represent the experimental range of replicate experiments (n = 2).

By comparing the ground truth AUC with the image-derived AUC for the corrected PET-CTs, we observe a very good agreement, overall, for the three tubes in the presence of background (Figure 9 dotted lines), having calculated AUC within 5 ± 3%, 8 ± 9% and 6 ± 14% of ground truth for the 1”,1/2” and 3/8” tubes, respectively. The peak heights in the presence of background were similarly accurate with values within 6 ± 2% (1”), 8 ± 5% (1/2”) and 9 ± 7% (3/8”) of the averaged DCE-CT curves. In the absence of background, the tubes are all somewhat less corrected in terms of AUC, 12 ± 2% (1”), 13 ± 3% (1/2”) and 22 ± 3% (3/8”). Under those conditions, the errors after correction in the peak heights, 9 ± 1% (1”), 2 ± 11% (1/2”) and 1 ± 12% (3/8”) are all relatively low.

### 3.4. PET-CT Results Corrections for Artery/Vein in Close Proximity

The previous Section 3.2 and Section 3.3 discussed only the 1”, 1/2” and 3/8” tubes due to the fact that the 1/4” input tube also contains a companion 1/4” venous flow tube. Due to this fact, using Equation 2 leads to an extreme overcorrection of the input function (Figure 9) in AUC of 139 ± 7% in cases with background and 152 ± 31% in cases without. This overcorrection occurs as a result of additional signal in the C_SO_/V_SO_ region coming from the venous tube, as well as the arterial tube. To account for this effect, Equation (6) must be employed instead in this scenario.

Using Equation (6) to address both the venous and arterial contributions to the “spill-out” region results in curves with AUC values within 13 ± 4% of the ground truth without background and 1 ± 17% with background. Similarly, the peak heights of the corrected 1/4” tubes are within 7 ± 5% of DCE-CT without background and 9 ± 10% with it. This is summarized in Table 1 and the results demonstrate that for all tube sizes down to 1/4”, and even in the presence of an alternative dynamic signal, it is possible to substantially correct the PVE in the small tube.

### 3.5. Application to Patient Scans

**Figure 10:**
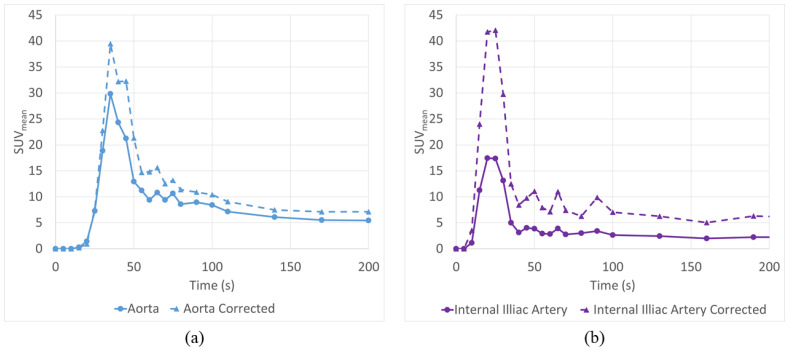
AIF Correction equations were successfully utilized to correct large vessels such as the descending Aorta (**a**) as well as smaller vessels such as the internal iliac artery (**b**) which is affected by both PVE and the neighboring internal iliac vein.

We applied the correction models to two patient dPET studies. Equation (2) was applied to the aorta (imaged ID 2.4 cm) of a patient undergoing dynamic PET-CT for a ^18^F-FAZA scan of the thorax, while Equation (6) was applied to correct the AIF from the internal iliac artery (IIA) (imaged ID 0.8 cm) in a prostate cancer ^18^F-FAZA PET-CT scan. Results of these corrections are displayed in Figure 10 (dashed lines).

As expected, the aorta correction was more moderate than the IIA, with a relative increase in AUC for the SUV_mean_ of only 25%, while the corrected IIA had an increase of over 150%.

## 4. Discussion

There are a number of PVE correction strategies available on modern PET-CT systems or their companion analysis workstations. Many of these correction strategies focus on producing an accurate SUV_max_ measurement for lesions, which can be reported and used to determine if a tumour is metabolically active or not. However, readings such as the SUV_max_, are strongly impacted by noise, due to the selection of only a single voxel [6,28]. This is especially problematic when performing dPET, as there is an inherent trade-off between signal to noise ratio and temporal resolution. In situations where kinetic modelling is to be utilized, the temporal resolution is critical, as setting the frame duration too long will result in decreased peak intensity and a flattened AIF. As such, frame times of 5–20 s are common, and this results in much noisier images than those observed in standard static PET, which have frame durations ranging from 2 to 5 min.

We have developed a relatively simple set of equations to correct for the PVE of AIF. The conservation of activity (CoA) correction method can be applied to any scanner, without the need for time of flight capability or built-in PSF correction algorithms. As the method only requires the ‘true’ geometry of the arterial region and dPET images, it is expected to work for PET-MR as well.

**Figure 11:**
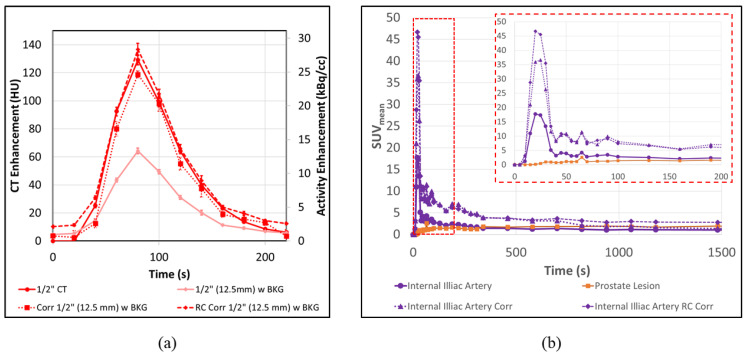
Correction of the AIF can be performed more accurately with the CoA based approach than with traditional recovery coefficient-based methods. (**a**) When applying a recovery coefficient-based correction the peak activity is recovered effectively; however, once the activity in the tubes drops below background levels, the effect of PVE is reversed leading to artificial increased activity levels. (**b**) This artificial increase in the AIF is apparent in patient scans where the RC-corrected AIF remains above tumour levels for the duration of the scan while the CoA method drops back below tumour levels around 800 s.

Other methods of PVE correction, such as those relying on recovery coefficients, will apply a linear correction to the ROI, based solely on size and regardless of the relative intensity of the background region and tube [1,6,23,24,25,26,27]. This may result in an AIF with a misrepresentative shape. Recovery coefficient (RC) -based methods are also incapable of dealing with situations involving multiple dynamic signals, as in the case of the internal iliac artery and vein. Figure 11a demonstrates a comparison between using a recovery co-efficient-based approach [23] (dashed line) and our CoA approach (dotted line) in the presence of background. Though the peak activity is recovered effectively using the RC method, once the activity in the tubes drops below background levels, the effect of PVE is reversed, leading to artificially increased activity levels. This is visualized in the last three points in the figure, where the CoA approach follows the DCE-CT scaled result, while the RC-based curve remains artificially increased.

^18^-FDG is much more accessible than ^18^-FAZA, which must be specially ordered. The use of ^18^-FDG or ^18^-FAZA is not expected to make a difference in the phantom scans, as both isotopes are ^18^-F based. The background concentration in the phantom (for our experiment with background) was selected based on the range of background tissue uptake at 60 min, observed in clinical studies with ^18^-FDG and ^18^-FAZA, according to our experience.

There is an inherent trade-off between signal to noise ratio and spatial resolution in PET imaging. It is interesting to see the effect of aliasing, as a result of 20 s frame duration, especially evident for the 3/8” tube. It is also interesting that the averaging of DCE-CT curves was able to predict the dPET curves with aliasing, accurately, when the dPET timing scheme was used. The averaged DCE-CT curves were chosen to be a reference for comparison with the corrected dPET curves. These two sets of curves matched very well in terms of shape and magnitude. The effect of the aliased AIF (as opposed to the ‘true’ AIF) on the accuracy of kinetic modeling is beyond the scope of this paper. 

As seen in Figure 8 and Table 1, the correction of the AIF is equally successful in conditions of background and without, when assessed by peak height. However, the correction in the non-clinical conditions, without background, do not appear to sufficiently correct for the full signal loss due to PVE. When examining the dashed line in Figure 8c, in particular, and to a lesser degree, Figure 8b, it appears that the points at the tail end of the curve are mostly to blame for the reduction in AUC. These points occur at a time when the AIF is well below 5 kBq/cc and, as they are not similarly underestimated in the presence of background, might be due to limitation of PET reconstruction for extremely low total counts.

In order for the assumption that the background contribution to the annulus of C_SO_ is represented by C_BKG_, it is important that the background region be sampled close to the “spill-out” region, but far enough not to be affected by the “spill-out”. Due to the constraint of fitting all five tubes in the PET-CT FOV, the tubes were placed about 2.5 inches (6.35 cm) apart. This physical limitation restricts how big the ROIs can be drawn around each tube before they start to encounter signal from neighbouring tubes. As a result, the background regions may include some minor “spill-out” from a neighbouring tube, which is not captured in the model. The impact of this in these experiments is an overestimation of the background signal, which leads to an underestimation of the AUC and peak of the CA_CORR_. This particular constraint did not occur in the patient examples. However, care should be taken when selecting the background region to avoid unaccounted areas of enhancement, contributed by nearby lesions or vessels. 

The proposed AIF correction method was applied to dPET studies of the thorax and pelvic area. As the two patient cases are of different patients, the results cannot be directly compared. However, the relative magnitudes of correction for aorta and IIA are 25% and 150%, respectively, similar to those in the phantom experiment for 1” and ¼” tubings. In addition, the corrected AIFs for the two cases have similar absolute magnitude, which is not unexpected for a fixed injection amount of radiotracer per patient weight. In both scenarios, the relative partial volume corrections of 25% and 150% to the aorta and IIA, respectively, are similar in impact to the phantom validation scans. It is also noted that the shape of the function is generally maintained, with the IIA taking on the classic arterial blood input function shape. 

Figure 11b demonstrates the difference between an RC-based correction of the AIF and our CoA-based approach. As in the phantom example, Figure 11a, once the activity in the AIF decreases below background levels, the RC-based approach tends to artificially overestimate the AIF, leading to curves, which might misrepresent the true physiology. In this example, the RC correction-based AIF (dashed line) continues to depict an activity greater than the prostate lesion for the duration of the scan, while the CoA-based approach (dotted line) drops back below tumour levels by about 800 s.

This is especially critical when performing kinetic modeling, as the shape of the input function is nearly as important as the absolute peak intensity in determining the model parameters [8,9,11]. Using the three AIFs and segmented tumour from the FAZA-Prostate patient example, shown in Figure 11b, an irreversible 2-tissue compartment model was fitted to the curves (PMOD software v3.5), with results displayed in Table 2. In this model, K_1_ represents the flow into a diffusive compartment, while K_2_ represents the reverse flow back to the bloodstream. K_3_ represents the flow into the accumulative compartment, which, in the case of FAZA, represents hypoxic regions.

Both the RC and CoA-corrected AIF result in a dramatic reduction of K_1_ (73.2% for RC and 70.6% for CoA) and K_2_ (43.0% for RC and 36.2% for CoA) when compared to the uncorrected AIF, as a result of the increased ratio between the magnitude of AIF and Tumour curves, and the remarkable difference in K_3_ highlights the advantage of the CoA-based correction over the RC method. Since the tail of the AIF curve always remains above the tumour curve, the model predicts a K_3_ of 0 for the RC-corrected AIF. Conversely, the model actually predicts a much larger K_3_ (363%) value for the CoA-based approach, suggesting an increase in hypoxia, as compared to the result when using the uncorrected AIF.

Though these results are promising with limited sample size, further investigation is required to validate the use of this method in patient studies.

## 5. Conclusions

We have presented a method for PVE correction for AIF in dPET, without the need for complicated PSF algorithms or advanced hardware. Our approach allows even dated systems without time-of-flight capability to perform dPET, and it is expected to produce improved quantitative results in kinetic models. The proposed method is less sensitive to noise compared to other PVE correction methods, as it uses much less sensitive SUV_mean_-based measurements. These equations were validated by a phantom and shown to be useful at correcting even 1/4” tubes by as much as 59 ± 35%; the correction leads to values within 8 ± 9% of the predicted values in the presence of background activity, in terms of AUC. The peak heights were similarly well corrected to within 9 ± 10% of the averaged DCE-CT.

We also demonstrated that the method can be applied to patient data, to generate more representative input functions for kinetic modelling of dynamic PET datasets.

## Figures and Tables

**Table 1:** Quantitative Results of Input Function PVE correction. (All results are shown ± the standard error of the mean n = 5.)

	1” (25.4 mm)	Cor. 1” (25.4 mm)	1/2” (12.7 mm)	Cor. 1/2” (12.7 mm)	3/8” (9.5 mm)	Cor. 3/8” (9.5 mm)	1/4” (6.35 mm)	Cor. 1/4” (6.35 mm)
No Background								
AUC (kBq/cc × s)	1399 ± 34	1827 ± 46	977 ± 39	1783 ± 68	747 ± 37	1610 ± 68	573 ± 31	1807 ± 87
% Error vs. Model	−33% ± 2%	−12% ± 2%	−53% ± 2%	−14% ± 3%	−64% ± 2%	−22% ± 3%	−72% ± 1%	−13% ± 4%
% Improvement vs. uncorrected		21% ± 3%		39% ± 4%		42% ± 4%		60% ± 8%
With Background								
AUC (kBq/cc × s)	1535 ± 39	1975 ± 72	1104 ± 48	1902 ± 183	941 ± 44	1950 ± 300	828 ± 42	2056 ± 351
% Error vs. Model	−26% ± 2%	−5% ± 3%	−47% ± 2%	−8% ± 9%	−55% ± 2%	−6% ± 14%	−60% ± 2%	−1% ± 17%
% Improvement vs. uncorrected		21% ± 4%		38% ± 9%		49% ± 15%		59% ± 35%

**Table 2:** Effect of AIF correction on kinetic model parameters.

	K_1_ (mL/cc/min)	K_2_ (1/min)	K_3_ (1/min)
Original AIF	0.1352	0.0965	0.0071
RC Corrected AIF	0.0363	0.055	0
CoA Corrected AIF	0.0398	0.0616	0.0328

## Data Availability

The phantom images created as part of this study will be made freely available upon publication.

## References

[1] Dimitrakopoulou-Strauss A., Pan L., Sachpekidis C. (2021). Kinetic modeling and parametric imaging with dynamic PET for oncological applications: General considerations, current clinical applications, and future perspectives. Eur. J. Nucl. Med. Mol. Imaging.

[2] Sokoloff L., Reivich M., Kennedy C., Des Rosiers M.H., Patlak C.S., Pettigrew K.D., Sakurada O., Shinohara M. (1977). The [^14^C] deoxyglucose method for the measurement of local cerebral glucose utilization: Theory, procedure and normal values in the conscious and anerstized albino rat. J. Neurochem..

[3] Wienhard K. (2002). Measurements of glucose consumption using [^18^F)fluorodeoxyglucose. Methods.

[4] Tomasi G., Turkheimer F., Aboagye E. (2012). Importance of quantification for the analysis of PET data in oncology: Review of current methods and trends for the future. Mol. Imaging Biol..

[5] Bettinardi V., Castiglioni I., De Bernardi E., Gilardi M.C. (2014). PET quantification: Strategies for partial volume correction. Clin. Transl. Imaging.

[6] Vallabhajosula S., Solnes L., Vallabhajosula B. (2014). A broad overview of positron emission tomography radiopharmaceuticals and clinical applications: What is new?. Semin. Nucl. Med..

[7] Phelps M.E., Huang S.C., Hoffman E.J., Selin C., Sokoloff L., Kuhl D.E. (1979). Tomographic measurement of local cerebral glucose metabolic rate in humans with (F-18)2-fluoro-2-deoxy-glucose: Validation of methos. Ann. Neurol..

[8] Carson R.E., Valk P.E., Bailey D.L., Townsend D.W., Maisey M.N. (2003). Tracer kinetic modeling in PET. Positron Emission Tomography: Basic Science and Clinical Practice.

[9] Bentourkia M., Zaidi H. (2007). Positron emission tomography. Tracer kinetic modeling in PET. PET Clin..

[10] Sachpekidis C., Eder M., Kopka K., Mier W., Hadaschik B.A., Haberkorn U., Dimitrakopoulou-Strauss A. (2016). ^68^Ga-PSMA-11 dynamic PET/CT imaging in biochemical relapse of prostate cancer. Eur. J. Nucl. Med. Mol. Imaging.

[11] Koukouraki S., Strauss L.G., Georgoulias V., Eisenhut M., Haberkorn U., Dimitrakopoulou-Strauss A. (2006). Comparison of the pharmacokinetics of ^68^Ga-DOTATOC and [^18^F] FDG in patients with metastatic neuroendocrine tumours scheduled for ^90^Y-DOTATOC therapy. Eur. J. Nucl. Med. Mol. Imaging.

[12] Henze M., Dimitrakopoulou-Strauss A., Milker-Zabel S., Schuhmacher J., Strauss L.G., Doll J., Mäcke H.R., Eisenhut M., Debus J., Haberkorn U. (2005). Characterization of 68Ga-DOTA-D-Phe1-Tyr3-octreotide kinetics in patients with meningiomas. J. Nucl. Med..

[13] Ilan E., Sandström M., Velikyan I., Sundin A., Eriksson B., Lubberink M. (2017). Parametric net imflux rate images of 68Ga-DOTATOC and 68Ga-DOTATATE: Quantitative accuracy and improved image contrast. J. Nucl. Med..

[14] Sachpekidis C., Afshar-Oromieh A., Kopka K., Strauss D.S., Pan L., Haberkorn U., Dimitrakopoulou-Strauss A. (2019). ^18^F-PSMA-1007 multiparametric, dynamic PET/CT in biochemical relapse and progression of prostate cancer. Eur. J. Nucl. Med. Mol. Imaging.

[15] Schmuck S., Mamach M., Wilke F., von Klot C.A., Henkenberens C., Thackeray J.T., Sohns J.M., Geworski L., Ross T.L., Wester H.J. (2017). Multiple time-point 68Ga-PSMA I&T PET/CT for characterization of primary prostate Cancer: Value of early dynamic and delayed imaging. Clin. Nucl. Med..

[16] Koopman T., Verburg N., Pouwels P.J., Wesseling P., Hoekstra O.S., De Witt Hamer P.C., Lammertsma A.A., Yaqub M., Wesseling P. (2020). Quantitative parametric maps of O-(2-(^18^F)fluoroethyl)-L-tyrosine kinetics in diffuse gliomas. J. Cereb. Blood Flow Metab..

[17] Kudomi N., Maeda Y., Hatakeyama T., Yamamoto Y., Nishiyama Y. (2017). Fully parametric imaging with reversible tracer 18F-FLT within a reasonable time. Radiol. Phys. Technol..

[18] Sachpekidis C., Thieke C., Askoxylakis V., Nicolay N.H., Huber P.E., Thomas M., Dimitrakopoulou G., Debus J., Haberkorn U., Dimitrakopoulou-Strauss A. (2015). Combined use of ^18^F-FDG and ^18^F-FMISO in unresectable non-small cell lung cancer patients planned for radiotherapy: A dynamic PET/CT study. Am. J. Nucl. Med. Mol. Imaging.

[19] Schwartz J., Grkovski M., Rimner A., Schroeder H., Zanzonico P.B., Carlin S.D., Staton K.D., Humm J.L., Nehmeh S.A. (2017). Pharmacokinetic analysis of dynamic ^18^F-Fluoromisonidazole PET data in non-small cell lung cancer. J. Nucl. Med..

[20] Grkovski M., Lee N.Y., Schroeder H., Carlin S.D., Beattie B.J., Riaz N., Leeman J.E., O’Donoghue J.A., Humm J.L. (2017). Monitoring early response to chemoradiotherapy with ^18^F-MISO dynamic PET in head and neck cancer. Eur. J. Nucl. Med. Mol. Imaging.

[21] Michaud L., Beattie B.J., Akhurst T., Dunphy M., Zanzonico P., Finn R., Mauguen A., Schöder H., Weber W.A., Lassman A.B. (2019). ^18^F-Fluciclovine (^18^F-FACBC) PET imaging of recurrent brain tumors. Eur. J. Nucl. Med. Mol. Imaging.

[22] Verwer E.E., Zegers C.M., van Elmpt W., Wierts R., Windhorst A.D., Mottaghy F.M., Lambin P., Boellaard R. (2016). Pharmacokinetic modeling of a novel hypoxia PET tracer [^18^F]HX4 in patients with non-small cell lung cancer. EJNMMI Phys..

[23] Feng S.T., Cui M., Gao J., Wu B., Sha W., Huang B. (2012). Image-derived arterial input function in dynamic positron emission tomography-computed tomography: A method using both positron emission tomographic and computed tomographic images. J. Comput. Assist. Tomogr..

[24] Anazodo U., Kewin M., Finger E., Thiessen J., Hadway J., Butler L., Pavlosky W., Prato F., Thompson T., Lawrence K.S. (2015). Preliminary evaluation of MRI-derived input function for quantitative measurement of glucose metabolism in an integrated PET-MRI. EJNMMI Phys..

[25] Muzi M., O’Sullivan F., Mankoff D.A., Doot R.K., Pierce L.A., Kurland B.F., Linden H.M., Kinahan P.E. (2012). Quantitative assessment of dynamic PET imaging data in cancer imaging. Magn. Reson. Imaging.

[26] Litton J.E. (1997). Input function in PET brain studies using MRdefined arteries. J. Comput. Assist. Tomogr..

[27] Croteau E., Lavallée E., Labbe S.M., Hubert L., Pifferi F., Rousseau J.A., Cunnane S.C., Carpentier A.C., Lecomte R., Bénard F. (2010). Image-derived input function in dynamic human PET/CT: Methodology and validation with 11C-acetate and 18F-fluorothioheptadecanoic acid in muscle and 18F-fluorodeoxyglucose in brain. Eur. J. Nucl. Med. Mol. Imaging.

[28] Lodge M.A., Chaudhry M.A., Wahl R.L. (2012). Noise considerations for PET quantification using maximum and peak standardized uptake value. J. Nucl. Med..

[29] Mourik J.E., Lubberink M., Klumpers U.M., Comans E.F., Lammertsma A.A., Boellaard R. (2008). Partial volume corrected image derived input functions for dynamic PET brain studies: Methodology and validation for [^11^C]flumazenil. Neuroimage.

[30] Cysouw M.C.F., Kramer G.M., Hoekstra O.S., Frings V., de Langen A.J., Smit E.F., van den Eertwegh A.J.M., Oprea-Lager D.E., Boellaard R. (2016). Accuracy and Precision of Partial-Volume Correction in Oncological PET/CT Studies. J. Nucl. Med..

[31] Chen K., Bandy D., Reiman E., Huang S.C., Lawson M., Feng D., Yun L.S., Palant A. (1998). Non invasive quantification of the cerebral metabolic rate for glucose using positron emission tomography, 18F-fluoro-2-deoxyglucose, the Patlak method, and an image-derived input function. J. Cereb. Blood Flow Metab..

[32] Zanotti-Fregonara P., Chen K., Liow J.S., Fujita M., Innis R.B. (2011). Image-derived input function for brain PET studies: Many challenges and few opportunities. J. Cereb. Blood Flow Metab..

[33] Driscoll B., Keller H., Coolens C. (2011). Development of a dynamic flow imaging phantom for dynamic contrast-enhanced CT. Med. Phys..

